# Validation of a Hybrid Exoskeleton for Upper Limb Rehabilitation. A Preliminary Study

**DOI:** 10.3390/s21217342

**Published:** 2021-11-04

**Authors:** Isabel-María Alguacil-Diego, Alicia Cuesta-Gómez, Aldo-Francisco Contreras-González, David Pont-Esteban, David Cantalejo-Escobar, Miguel Ángel Sánchez-Urán, Manuel Ferre

**Affiliations:** 1 Physiotherapy, Occupational Therapy, Physical Medicine and Rehabilitation Department, Universidad Rey Juan Carlos, Campus de Alcorcón, Av. de Atenas, s/n, 28922 Alcorcón, Spain; isabel.alguacil@urjc.es (I.-M.A.-D.); alicia.cuesta@urjc.es (A.C.-G.); 2 Centro de Automática y Robótica (CAR) UPM-CSIC, ETS Ingenieros Industriales, Universidad Politécnica de Madrid, Calle de José Gutiérrez Abascal, 2, 28006 Madrid, Spain; af.contreras@alumnos.upm.es (A.-F.C.-G.); david.pont@upm.es (D.P.-E.); david.cantalejo@upm.es (D.C.-E.); miguelangel.sanchezuran@upm.es (M.Á.S.-U.); 3 ETS Ingeniería y Diseño Industrial, Universidad Politécnica de Madrid, Ronda de Valencia, 3, 28012 Madrid, Spain

**Keywords:** rehabilitation, upper-limb, exoskeleton, robot

## Abstract

Recovery of therapeutic or functional ambulatory capacity in patients with rotator cuff injury is a primary goal of rehabilitation. Wearable powered exoskeletons allow patients to perform repetitive practice with large movements to maximize recovery, even immediately after the acute event. The aim of this paper is to describe the usability, acceptability and acceptance of a hybrid exoskeleton for upper-limb passive rehabilitation using the System Usability Scale (SUS) questionnaire. This equipment, called ExoFlex, is defined as a hybrid exoskeleton since it is made up of rigid and soft components. The exoskeleton mechanical description is presented along with its control system and the way motion is structured in rehabilitation sessions. Seven patients (six women and one man) have participated in the evaluation of this equipment, which are in the range of 50 to 79 years old. Preliminary evidence of the acceptance and usability by both patients and clinicians are very promising, obtaining an average score of 80.71 in the SUS test, as well as good results in a questionnaire that evaluates the clinicians’ perceived usability of ExoFlex.

## 1. Introduction

Alterations of the musculoskeletal system are one of the conditions of greater medical consultation at different levels of health care, and within these, shoulder pain occupies an important place, also causing considerable functional disability to perform activities of daily life [1]. The rehabilitation programs for pathologies of trauma origin at the level of the upper limb have as common objectives the increase of the joint range to promote the highest level of functionality of the patients.

In some pathologies of traumatic origin such as rotator cuff tendinopathy or frozen shoulder, recovery of functional capacity must begin early. Though, in others, such as fractures, dislocations and arthroplasties, it is important to respect an initial period of immobilization, which helps recovery, reduces pain and edema, helps consolidation and prevents radiological deformities [2]. After this period of immobilization, it is necessary to restore the patient’s functionality using various techniques such as kinesitherapy as soon as possible because of complications such as joint stiffness, muscle atrophy, bone degradation and both capsular and ligamentous retraction, which the immobilization produces [3]. However, it is not always possible to meet these demands for early and intensive care, especially in the current situation due to the COVID-19 epidemic, where the time to start treatments has been increased due to the limitation/restriction of the number of patients in physiotherapy rooms and the possibility of being treated manually by the therapist, it being frequent to refer the patient to his home with an exercise regimen that did not always present the required adherence, either due to lack of time, lack of motivation or due to pain.

Exoskeleton-assisted rehabilitation has become increasingly popular in recent years and the use of exoskeletons in the field of rehabilitation has been a real hit [4]. Exoskeletons assist therapy by providing several advantages over conventional approaches, including a standardized training environment, adaptable support and the ability to increase treatment intensity and dose, while reducing the physical burden on therapists. Exoskeleton-based rehabilitation systems provide a solution to increase the number of movements, involve safe and intensive rehabilitation exercises and have the advantage that the patient’s movements can be measured objectively [5]. Rehabilitation exoskeletons are, therefore, an ideal means of complementing conventional therapy in the clinic, presenting great potential for continuing therapy and care at home using simpler and more portable devices [6].

The acceptance of exoskeletons has been increasing over the years, although, despite the advantages offered by these devices and the great variety on the market, they are not yet fully implanted in the clinic as a complementary tool in rehabilitative therapy [7]. The importance of knowing the opinion of users, both clinicians and patients, should be noted. Therefore, it is essential to evaluate the experience of both of them with the exoskeletons in terms of usability, acceptability and acceptance, in the process of developing an exoskeleton device. According to a definition by the International Organization for Standardization, usability refers to the effectiveness, efficiency, and user satisfaction rating of a product in a specific environment by a specific user for a specific purpose. It includes three aspects: effectiveness (i.e., the accuracy and completeness of a goal that is achieved by a product); efficiency (i.e., the effort required for a user to complete a task); and satisfaction (i.e., the comfort and acceptability of a product). Usability tests and evaluations aim to make medical equipment easier, safer, and more effective and pleasant for users. A usability evaluation helps wearable devices to satisfy the requirements of the market and consumers. In this regard, one of the most popular tools for evaluating the usability, acceptability and acceptance [8] of exoskeletons is the System Usability Scale (SUS).

The remainder of this paper is structured as follows. Section 2 describes ExoFlex and the movements that it can assist in rehabilitation. In Section 3, SUS and the clinician’s questionnaire are presented. Section 4 gathers the validation results for both tests. In Section 5, a discussion about the results and a comparison with other devices is performed. Finally, Section 6 gathers the main conclusions of this work.

## 2. Materials and Methods

**Figure 1:**
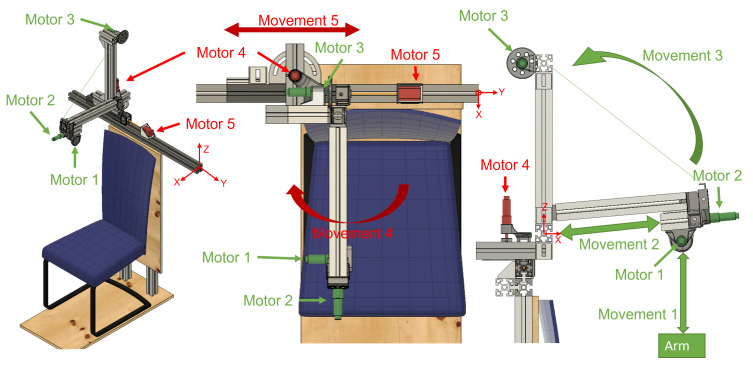
Views of the hybrid exoskeleton developed for arm rehabilitation treatments. Active joints during patient rehabilitation are shown in green; joints for the adjustment of the structure are shown in red. The origin of the system is marked in the three views.

ExoFlex is a cable-driven hybrid exoskeleton intended for upper-limb passive rehabilitation. This kind of rehabilitation is usually used in the early stages of the rehabilitation process, and it is based on moving the subject’s limb while they leave it completely relaxed [9]. A nylon-covered steel cable is attached to the subject’s arm via a flexible wearable textile coupling. The other end of the cable is attached to a pulley inserted in the shaft of motor m1 (Figure 1), which is coupled to the end effector of the exoskeleton. In this way, the exoskeleton is capable of moving the user’s arm by controlling the length of the cable (winding or unwinding it over the pulley) and by properly positioning the rigid structure’s end effector. This exoskeleton is considered hybrid since it is made up of a mix of rigid components fixed to the floor and a wearable coupling worn by the patient. The rigid part not only accounts for the precise position of the end effector of the exoskeleton, but additionally serves to locate the control electronics.

With regard to the methods used for the evaluation of ExoFlex, we have used the SUS to evaluate the patients’ acceptance of the device and an additional questionnaire for the clinicians in order to evaluate the perceived potential of this tool for rehabilitation. Both are presented in the following section.

### 2.1. Exoskeleton Overview

The exoskeleton includes five Degrees of Freedom (DoF). The actuation system is constituted by four DC motors (motors m1, m2, m3 and m4) and motor m5 is a Longruner Nema-17 LD08 stepper motor. The DC motors are DCX22S GB KL 48V motors with planetary reducers with reduction ratios of 794, 14, 326 and 794 for motors m1, m2, m3 and m4, respectively. These four motors include high resolution ENX16 EASY 1024IMP encoders. Lead screws have been attached to the shafts of motors m2 and m5 in order to transform rotational movement into linear displacement.

Each ExoFlex joint is endowed with an optical limit switch (Omron EE-SX4009-P1). Each time ExoFlex is turned on, a calibration process is performed. This consists of a sequential movement of all the motors at a predefined speed until each joint achieves its limit, signaled by each limit switch. These sensors also work as a safety system.

The LAUNCHXL-F28379D dual-core 32-bit micro controller from Texas Instruments has been used to interface with the actuation system and to implement low-level control. A  Printed Circuit Board (PCB) called ALICE (*Assistive LImb Control Electronics*) has been specifically designed for the exoskeleton. It contains all the necessary hardware (current sensors, ADCs, communication interfaces and drivers for motors) in a compact and modular way. This shield-style board is mounted on top of the microcontroller board. The control of the motors is performed with a super-twisting sliding mode controller (SMC). The use of a robust controller is mandatory in this kind of applications, where there appear many unmodelled phenomena, such as slacks and hysteresis in the exoskeleton’s fabric; and there can exist significant variability among users in terms of anatomical parameters. Robustness to external disturbances is also critical. More details are available in Appendix B. For safety purposes, two emergency buttons have been allocated in the chair, one at each side. They can be pressed at any time by the patient or the therapist to immediately stop the rehabilitation and move the robot to the initial position.

The used encoders are of the quadrature-type and have a high resolution (1024 pulses per revolution). The counting of the pulses must be carried out by means of a hardware counter, since the high frequency of reception of pulses does not allow counting by means of interruptions. The LAUNCHXL-F28379D board has embedded three hardware counting modules for encoders, which are used for motors m1, m2 and m3. An external pulse counting module (LS7366R) has also been added for m4. This module communicates with the microcontroller through SPI and greatly lightens the software load of the microcontroller. Motor m5 position is obtained by counting the number of steps.

**Figure 2:**
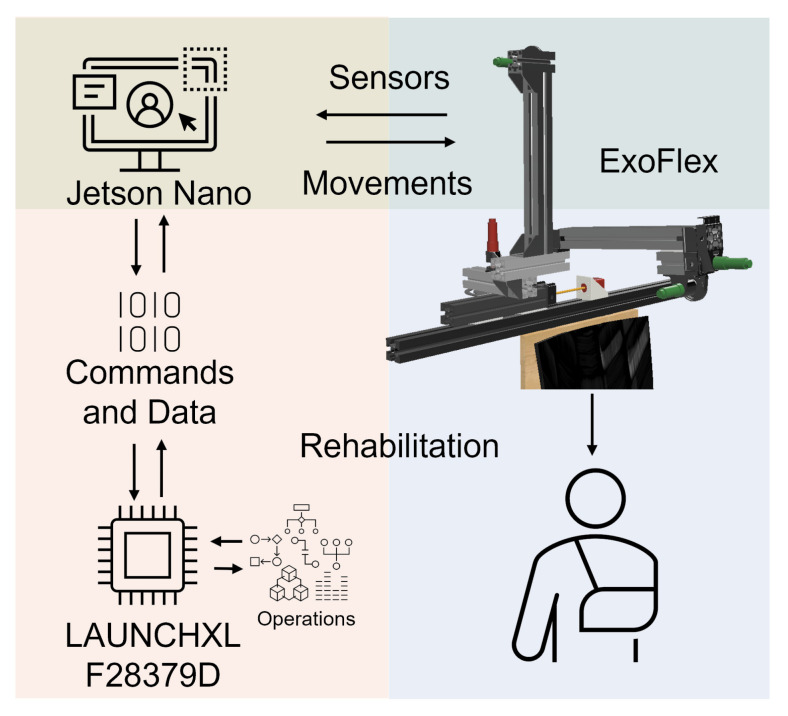
System architecture. Electronics, actuators and control.

A multi-threaded real-time application has been developed to control the exoskeleton. In this way, we have precise control over the frequency with which data from the different sensors is obtained, the control period and the speed of the communications. The design of the application is modular, which makes it easy to adapt the system to new needs that may arise during the development of the device (see Figure 2).

A Jetson Nano communicates with the microcontroller via SPI to transmit movement references and store the data obtained during work sessions for later analysis. A Graphical User Interface (GUI) has been implemented on the Jetson Nano to be able to interact in a simple and friendly way with the exoskeleton and monitor the data in real time. It allows us to select rehabilitation session options. In each session, the type of movement, the elevation angle and speed, the number of repetitions and a waiting time between movements can be configured. The configuration determines the commands to be sent to the motor controllers at any time. The fact that the electronics and software are our own designs allows us to obtain an architecture highly adapted to the needs of the exoskeleton.

Exoskeleton motion generation is produced as follows. Motor m1 controls the length of the cable. Motor m2 generates linear displacement of the end effector of the exoskeleton along the rod to which it is attached while motor m3 allows performance of the elevation of that rod by winding the cable anchored to it over its pulley. Motor m4 controls the rod angle with respect to the sagittal plane (we call this angle shoulder aperture angle θ) and motor m5 horizontally moves all the actuation structure to accommodate the user’s complexion. On the one hand, motors m1, m2 and m3 are active during the rehabilitation therapies to generate the predefined patient arm movement. On the other hand, actuators m4 and m5 are only used for adjusting the mechanical structure to the kind of treatment and patient body size (see Appendix C for further details).

**Figure 3:**
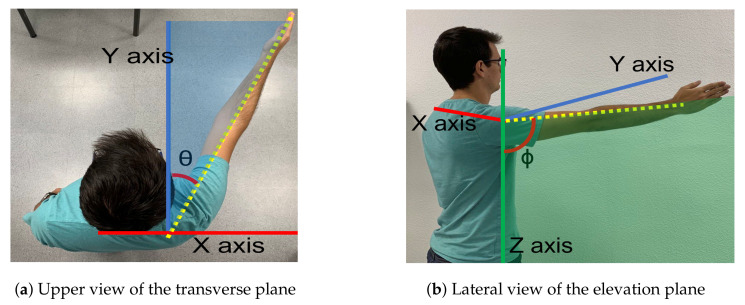
Motion planes. (**a**) The angle (θ) between the arm and the sagittal plane along the transverse plane determines the elevation plane. (**b**) The angle ϕ is the elevation angle of the arm in the elevation plane.

The exoskeleton range of motion for arm elevation angle ϕ is comprised between 0 degrees and 160 degrees. That elevation range is guaranteed for every shoulder aperture angle θ between 0 degrees (pure flexion) and 90 degrees (pure abduction). Figure 3 shows those two angles involved in shoulder motion for a concrete pose.

The wearable coupling between the user and the rigid structure through the transmission cable is an adjustable piece of fabric that is placed on the wearer’s limb and distributes pressure across different specially designed seams. More specific details about its design can be found in Appendix A. The main purpose of an exoskeleton is to achieve all joint rotations and the alignment of the exoskeleton joints with the anatomical ones. The misalignment of the axes and the two additional translation DOF in the human shoulder require additional mechanisms, making the exoskeleton more complex to build and use [10]. Lightweight aluminum profiles have been used to build the fixed structure of the exoskeleton in a modular fashion. Custom parts necessary for the assembly of the whole structure were designed and 3D printed with a material which combines nylon with carbon fiber. A static stress study for the structure was performed via simulation and a safety factor of 2.66 was obtained for a force of 100 N applied on the tip of the rod where motor m2 is placed, which is the point where the applied force generates more torque. The structure is designed to increase the height of the entire system manually in case any subject needs it.

### 2.2. Setup Description for Rehabilitation

At the beginning of the session, the subject sits down in the chair and the position of the acromioclavicular joint is measured with respect to a reference point of the exoskeleton. This process is carried out only once since the user data are stored on the interface for future sessions.

**Figure 4:**
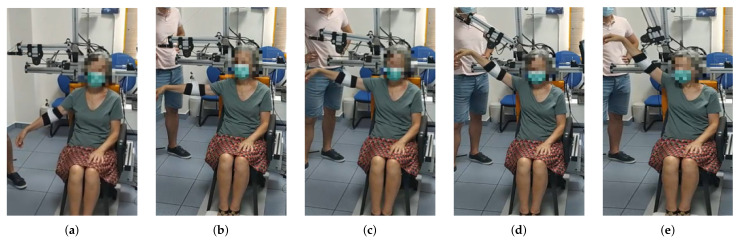
From (**a**–**e**) a series of frames corresponding to an abduction movement ($\theta =90^{\circ }$) increasing the angle ϕ are shown. Note that the exoskeleton end effector is not moved until the subject´s arm is at 90 degrees. From then on, motors m2 and m3 move synchronously in order to properly move motor m1 in the workspace while the cable length is being controlled.

**Figure 5:**
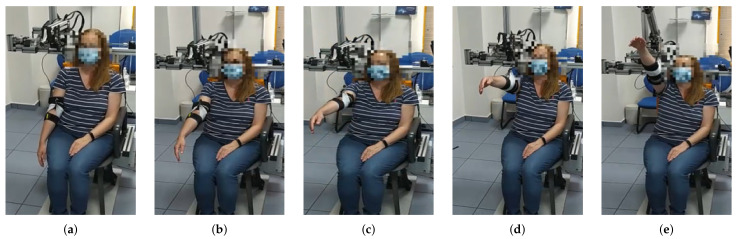
From (**a**–**e**) a series of frames corresponding to a flexion movement ($\theta =0^{\circ }$) are shown. Similarly to the abduction movement, until reaching the 90 degrees arm elevation, the elevation of the arm is uniquely performed by reducing the cable length. In this case, a maximum elevation angle ϕ of 130 degrees was programmed, according to the patient’s needs.

**Figure 6:**
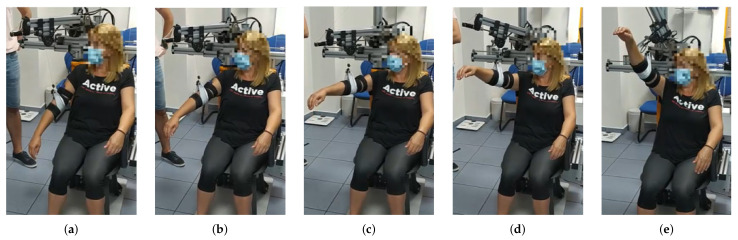
From (**a**–**e**) a series of frames corresponding to an intermediate aperture angle elevation ($\theta =30^{\circ }$) are shown. The maximum elevation angle for this patient was set to $\phi =145^{\circ }$, according to the clinicians’ indications.

The session is set out as follows: the therapist determines the shoulder’s aperture angle θ with which the lifting movement is desired to be performed. Once the aperture angle has been selected, motors m4 and m5, according to the measured position of the subject’s shoulder, are positioned so that the mobile rod is parallel to the arm and the end effector (pulley of motor m1) is aligned with the arm ( Algorithm 1). These motors (m4 and m5) will not make any movement during the arm lift. Afterwards, the shoulder maximum elevation angle ϕ, the movement speed, the number of repetitions and the pause time between consecutive repetitions are selected. During both the ascending and descending stages of the movement, motor m1 controls the cable length and motors m2 and m3 are in charge of mobilizing the exoskeleton end effector according to the specified range of motion. Given that the main pulley of motor m1 is positioned in the form of a crane, if the elevation angle of the arm is less than 90 degrees, motors m2 and m3 will not move in the session ( Algorithm 2). In the case where the angle is greater, those two motors will move synchronously to rise the arm beyond that threshold. This behavior can be observed in Figure 4, Figure 5 and Figure 6 and the algorithm is described in the Appendix D.

### 2.3. Subjects

A total of seven participants (six women and one man) with upper limb impairments participated in the study. The age of the patients ranged between 50 and 79 years (63.57 ± 10.72 years). The sociodemographic characteristics and the health status of the participants in the study is summarized in Table 1.

The inclusion criteria established for the selection of the patients were: presentation of musculoskeletal injury of the right upper limb, being a subsidiary of rehabilitative treatment, not presenting any pathology that contraindicates the rehabilitative treatment and ages over 18 years. Patients who presented some of the following exclusion criteria were not admitted to the study: cognitive impairment that implies not understanding simple commands, neurological injury that affects the upper limb, and dermatological injury that prevents the use of exoskeleton material.

This protocol was approved by the Research Ethics Committee of the Rey Juan Carlos University. The ethical principles for medical research in humans of the Declaration of Helsinki were followed. All subjects signed the informed consent before participation. The trials were carried out at the Clinic Center of Getafe (Madrid, Spain). Before performing the device test, the patients provided sociodemographic data and a rehabilitation doctor evaluated their health status. The test consisted of a series of passive mobilizations of the affected upper limb: shoulder flexion, shoulder abduction and shoulder elevation with an intermediate aperture angle. These movements are the same as those performed by physiotherapists in conventional physiotherapy sessions.

All tests were performed in the patients’ painless range of motion (ROM). ROM was evaluated by a doctor before performing the test. The active range of movement of the patients was determined, as well as the ROM, in which the patient could perform the movement without pain, and thus the exact angles at which the exoskeleton would perform the movements were determined.

## 3. Usability and Acceptance Scales

ExoFlex performance has been evaluated from the point of view of the patient and the point of view of the clinician. The next subsections show the details of each questionnaire.

### 3.1. System Usability Scale Questionnaire

After testing ExoFlex, the participants completed the SUS questionnaire that collected subjective evaluations and recommendations regarding the device (Table 2). The questionnaire has been translated from Spanish to English, since the patients were Spanish-speaking, and the questionnaire was in their language. The SUS was developed by Brooke [11] as a system usability tool, which has been widely used in the evaluation of a range of systems [12].

The SUS provides a quick and reliable tool for measuring the usability of a device. Furthermore, the SUS is versatile and can be used to evaluate websites, software, mobile devices, and medical systems; it is a short questionnaire that is quick to answer; a final score is provided with an interpretation based on a well-established reference standard; it is free, it is suitable even when applied to small samples (N < 14) and it has excellent reliability (0.85). Overall, the SUS is a quick and simple method for usability evaluation. It consists of a 10-item questionnaire with five response options for respondents; from strongly agree (score of 5) to strongly disagree (score of 1). There are five positive statements and five negative ones, which are presented in alternation. Odd numbered questions (Q1, 3, 5, 7, and 9) were positive questions, and the recorded scores were the original scores subtracted by 1. Even numbered questions (Q2, 4, 6, 8, and 10) were negative questions, and the recorded scores were 5 minus the original ones. Once the results for the ten questions are treated, the score of each question is added and the result is multiplied by a 2.5 factor. The score of the SUS, therefore, ranges from 0 to 100, where a higher score means better usability, with a threshold of 68 that determines the usability of the device.

### 3.2. Clinician Questionnaire

We have evaluated clinicians’ perceived usability, acceptability, and satisfaction of ExoFlex by a satisfaction questionnaire. Five items are rated on a Likert-type scale from 1 to 5 (strongly disagree—strongly agree) [13]:

1. “Are you satisfied with ExoFlex?”;
2. “Has ExoFlex been useful for the rehabilitation of the upper limb?”;
3. “Do you think that ExoFlex could be helpful in the process of upper limb rehabilitation?”;
4. “Would you recommend ExoFlex to other clinicians?”;
5. “Do you think ExoFlex has advantages compared to other devices?”

The arithmetic mean across all items provides the total satisfaction score.

## 4. Results

Results on the patient’s opinion on the usability and acceptability of the exoskeleton and clinicians’ evaluation of the usefulness of the device for rehabilitation therapies are presented in the following subsections.

### 4.1. Patients Acceptability Assessment

**Figure 7:**
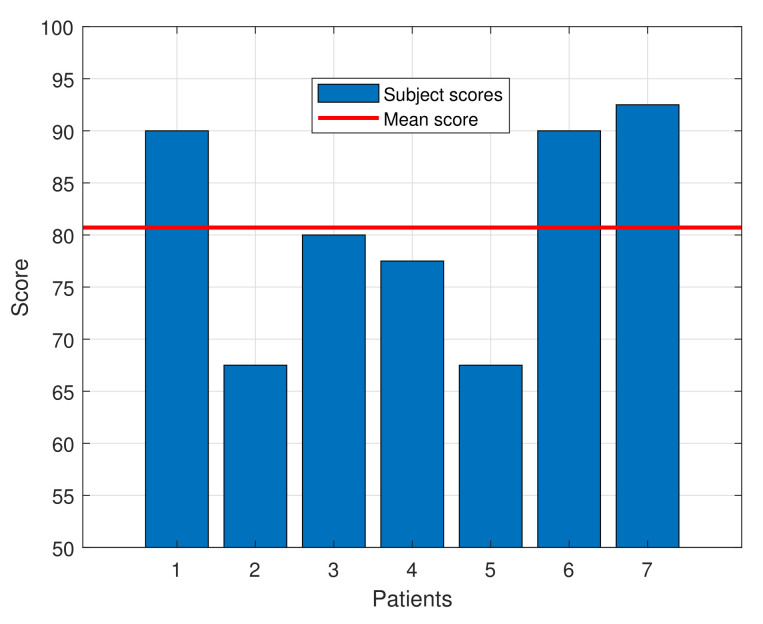
SUS score for each patient along with the mean score.

The SUS questionnaire score for the subjects ranged from 67.5 to 92.5, with an average score of 80.71 ± 9.79, indicating a high degree of acceptance by the patients. That score exceeds the average of 68, which, according to the SUS questionnaire standards, defines a tool as usable. Moreover, as defined in [14], a device with this score can be classified as “Excellent” in the acceptability range. Table 3 shows the average score for each question of the SUS questionnaire and Figure 7 presents a bar graph with the individual SUS score for each patient and the average one.

Among the results, some aspects should be highlighted. A result of 3.4 in each question would lead to obtain a SUS score of 68. Questions Q1, Q3, Q5, Q7, Q9 and Q10 widely surpass that threshold, while the other questions’ averages do not meet it. The results in question Q9 show that all the patients felt completely safe during the tests. Given that the average age of the participants is quite elevated, this result is even more valuable, since older people are normally more frightened and reluctant to try new technologies than youngsters. Finally, question Q4 is the one with lowest score, showing the almost unanimous perception that the participants feel the need to be helped by a person with technical knowledge about the device.

Apart from the SUS questionnaire, the participants were also asked about any adverse effects that may have happened during the trial and no relevant adverse effects were reported. It is important to say that one of the patients stated that she felt less pain when the device performed the movement than when the therapist performed it in her regular therapy, both reaching the same maximum elevation angle.

### 4.2. Clinician’s Satisfaction Assessment

The perceived usability, acceptability, and satisfaction of ExoFlex by clinicians is summarized in Table 4. The responses of the clinicians who participated in all the tests performed by the participants showed a high degree of satisfaction with the device. The average satisfaction score for the clinicians was 4.0, which corresponds with an overall “agree”.

The two clinicians evaluated “strongly in agreement” with satisfaction, usefulness and help in the rehabilitation process, one of the clinicians evaluated “strongly agree” with the possibility of recommending it to other clinicians, nevertheless the other scored “agree”. However, all participants stated “neutral” with the advantages of using ExoFlex compared with other exoskeletons, perhaps due to the lack of knowledge about the rest of the robotic devices for the upper limb in terms of price and benefits for patients.

## 5. Discussion

The present study shows the use of a hybrid exoskeleton system for upper limb passive rehabilitation, and describes the preliminary evidence of usability, acceptability, and acceptance by patients and clinicians.

In order to evaluate the grade of acceptance of this kind of technology, the most common scales used include the SUS, the ISONORM 9241/10 Questionnaire, and the Post-Study System Usability Questionnaire (PSSUQ), being the most popular the SUS [15]. It is worth highlighting the score of 80.71 ± 9.79 obtained in the SUS of this study, which indicates a high degree of acceptance. In fact, this score can be classified as “Excellent” in the acceptability range. In addition, it should be noted that the clinicians were highly satisfied with the device, as their responses to the clinicians’ questionnaire show. Also, at this point it is good to mention that this type of questionnaire uses ordinary scores which some authors see as a bad practice [16] and could make an approximation to the measurements by interval [17].

The acceptability of similar devices has been evaluated using the SUS. For instance, a motorized exoskeleton interface [18] obtained a “Good” overall score evaluated on 11 subjects (6 injured and 5 healthy) while [19] has tested a ExoGlobe with 14 experienced occupational therapists, obtaining a score of 63.75. A passive exoskeleton for static upper limb activities [20] got a score over 70, therefore being deemed to be usable. Tests were performed with eight participants. Additionally, in [21], the SUS score obtained from the patients, caregivers, and therapists (a total of 17 subjects) was 71.8 ± 11.9, indicating a high level of usability and product acceptance. The statistical assessment for a robot-based tool used on rehabilitation [22] has shown a mean score of 79.3 testing with seven injured patients.

For some exoskeletons, the mean score rises above 90, as the case of an assistive device for upper limb support [23] tested with users affected by neuromotor disorders. As an exceptional case, but not for shoulder rehabilitation, we can find the DexoHand [24] which has obtained in the SUS a score, for both the patients and healthy subjects, of 94.77 ± 2.98, indicating an excellent level of usability. On the other hand, authors in [25] have evaluated two behavior methods on the same device, one using muscle activity and the other one, manual control. They found out that the manual control has gotten a score of 77 but the one using the muscle activity has no acceptance score.

In the case of exoskeletons for lower extremities [26] completed two user experience questionnaires, the Quebec User Evaluation of Satisfaction with assistive Technology (D-QUEST) and the SUS. Participants were satisfied with the exoskeleton (D-QUEST 3.7 ± 0.4) and the SUS score was 72.5.

As has been exposed, several of these studies have applied the SUS to patients and healthy people, including in some cases therapists. Others have only applied the SUS to patients. As the latter ones, we have decided to apply the SUS only to patients since these are the ones that are going to use the exoskeleton in their own body, so their acceptability score is the important one, in comparison to the one of healthy subjects or therapists. Similar to some of the aforementioned studies, we consider that the clinician’s opinion about the usefulness of the exoskeleton is extremely important. However, in order to evaluate it, we considered that the inclusion of a specific questionnaire for them was of great value, since more concrete aspects can be evaluated. As we believe, applying the SUS questionnaire to clinicians provides unavailing information in comparison to the one obtained by the specific clinicians’ test that has been designed. In this way, we have two different metrics for the evaluation of the acceptability of very different groups—the patients and the clinicians.

### Study Limitations

The current study has several limitations. First, the sample of subjects was small. Therefore, caution must be taken in generalising the results. Second, our study did not evaluate whether scores obtained with ExoFlex were clinically significant. Third, only two clinicians evaluated the device’s usability, acceptability, and satisfaction. Further studies are necessary to examine ExoFlex’s impact in rehabilitation outcomes in treating upper limb pathologies.

The limitations of the ExoFlex as a rehabilitation tool can be listed as follows:

1. The maximum angle of elevation of the subject’s arm is 160 degrees;
2. This version of the ExoFlex does not perform shoulder rotations;
3. The trajectory generation is focused on arm lifting movements in a fixed plane. Further work will focus on varying the movement plane during the elevation process.

## 6. Conclusions

The validation of the usability and acceptability for both patients and clinicians with respect to the upper-limb passive rehabilitation exosuit ExoFlex has been addressed. In the preliminary results, the average SUS score for the patients was 80.71 ± 9.79, far surpassing the threshold of 68 necessary for considering the device as acceptable to the patients. Moreover, the ExoFlex score in SUS is comparable that of other state-of-the-art exoskeletons, even improving the scores obtained by several of those presented in the Discussion section.

Regarding the clinicians’ satisfaction with the tests, although they positively valued the exoskeleton in terms of usefulness and satisfaction, they could not make a judgment about the advantages or disadvantages of ExoFlex in comparison with other similar devices in rehabilitation therapies. For this purpose, parallel tests should be performed with ExoFlex and other existing options. Finally, to further characterize the potential of this exoskeleton, the following tests will involve middle or long-term therapies in order to obtain quantitative results about the benefits of using ExoFlex in the full treatment of a patient.

## Figures and Tables

**Table 1:** Participant profile.

Participant	Sex	Age	Upper Limb Pathology	Experience in Using Rehabilitation Exoskeleton
1	Male	78 yrs	Shoulder surgery (Supraspinatus suture)	No
2	Female	60 yrs	Troquiter Fracture plus tendinosis supraspinatus	No
3	Female	65 yrs	Supraspinatus partial tear plus frozen shoulder	No
4	Female	79 yrs	Supraspinatus tear	No
5	Female	51 yrs	Rotator cuff tendinosis	No
6	Female	62 yrs	Rotator cuff tendinosis	No
7	Female	50 yrs	Supraespinatus tendinosis	No

**Table 2:** System Usability Scale questionnaire.

Item	Question
Q1	I think I would use this device frequently
Q2	I find this device unnecessarily complex
Q3	I think the device was easy to use
Q4	I think I would need help from a person with technical knowledge to be able to use this device
Q5	The functions of this device are well integrated
Q6	I think the device has a lack of consistency
Q7	I imagine that most people would learn to use this device very quickly
Q8	I find the device very difficult to use
Q9	I feel safe using this device
Q10	I needed to learn many things before being able to use this device

**Table 3:** Results of the Usability Scale questionnaire for each question. The score is expressed as mean ± standard deviation.

Question	Score
Q1	3.57 ± 0.73
Q2	3.43 ± 0.90
Q3	3.43 ± 0.49
Q4	1.57 ± 1.18
Q5	3.29 ± 1.03
Q6	2.86 ± 1.46
Q7	3.14 ± 1.36
Q8	3.14 ± 1.12
Q9	4.00 ± 0.00
Q10	3.86 ± 0.35
**TOTAL**	**80.71 ±9.79**

**Table 4:** Results of the clinician assessment.

Clinical	Item	Strongly Disagree	Disagree	Neutral	Agree	Strongly Agree
1	1					X
	2					X
	3					X
	4					X
	5			X		
2	1					X
	2					X
	3					X
	4				X	
	5			X		

## Data Availability

Not applicable.

## Appendix A

The research team has previously proposed a body-adaptable textile design that aids performance for shoulder movements by combining textile layers with force-fit seams. This design also includes pieces for guiding, anchoring and cable support. These pieces use different methods of construction to facilitate manufacture, use and cleaning [27]. On the other hand, the team has developed a wearable wired and textile exoskeleton to help flex the elbow and shoulder. In this docking interface, different fabrics and sewing patterns are combined to promote force distribution and adaptation to the user’s anatomy [28]. These textiles are used in this research.

## Appendix B

A super twisting slider mode controller [29] has been simulated and implemented previously for elbow and shoulder flexion and extension movements [30]. In the study conducted previously by the authors, the use of a supertwisting controller is proposed, which stands out from other controllers in sliding mode due to its attenuation of chattering, green a high frequency noise coupled to the control signals due to the discontinuous nature of SMC. This controller is applied to the four DC motors of the system. The control law for motor *i* is given by ((A1)–(A4)):

(A1) $$u_{i}=c_{i}{\left|\sigma_{i}\right|}^{\frac{1}{2}}sign\left(\sigma_{i}\right)+w_{i}$$

(A2) $${\dot{w}}_{i}=b_{i}sign\left(\sigma_{i}\right)$$

(A3) $$c_{i}=1.5\sqrt{C_{i}};b_{i}=1.1C_{i}$$

(A4) $$\sigma_{i}=\sigma_{i1}e_{i1}+\sigma_{i2}e_{i2}+\sigma_{i3}e_{i3}$$

where, for the motor *i*, $u_{i}$ is the control action, $\sigma_{i}$ is its sliding surface (given by the coefficients $\sigma_{ik}$), $\omega_{i}$ is the term for the attenuation of the chattering, $C_{i}$ is a control parameter and the $e_{ij}$ are the errors associated with the motor state reference *i* consisting of the position, speed and current of it. The horizontal positioning stepper motor of the structure is controlled in an open loop, which is perfectly feasible since both the speed and the torque to be applied by the motor are low enough so that no step is lost.

## Appendix C

There are two types of synchronized movements to perform the subject’s shoulder lift. The first relationship of movements is the one that occurs with motors m4 and m5 in order to reach the work plane. The working plane is calculated by obtaining the distances in X, Y and Z from the zero of the structure (see origin at Figure 1), to the subject’s shoulder, explicitly at the acromioclavicular joint. Detection of this junction is accomplished by touching the upper shoulder between the deltoid and trapezius muscles. Once the distances are obtained calculations are made in order to locate the motors as developed on the Algorithm A1.

The aperture angle defines the angle of the motor m4 while the motor m5 moves linearly searching a parallel relationship with the subject’s arm. On this method the maximum distance achievable by each motor (m4_limit and m5_limit) and a fixed distance of the robot ($robot_{bar}$) are used.

The second movement relationship is the one that produces the elevation of the shoulder using the motors m1, m2 and m3 as developed on the Algorithm A2.

This movement consists of three stages of the behavior of these motors. The first stage is the elevation of the motor m1 to a certain threshold, at this moment m1 stops and the motor m3 begins to move (stage two), the motor m4 is activated in a third stage without stopping the motor m3. In this way, a continuous movement of the arm is caused and a perpendicularity between the cable and the arm is achieved, which causes better performance.   

**Algorithm A1:** Relationship of the aperture angle with the displacement of the motors m4 and m5
**Data**: θ, m4_limit, m5_limit, $usr_{X}$, $robot_{bar}$ **Result**: Motor 5 displacement distance motor_4=θ; /*( angle of motor m4 is defined by θ( */ $p2p1=m5\_limit*\frac{\theta }{m4\_limit}$; /*( calculated distance ( */ $motor\_5=usr_{X}+robot_{bar}-p2p1$; /*( displacement distance ( */

**Algorithm A2:** Relationship of the aperture angle with the displacement of the motors m1, m2 and m3

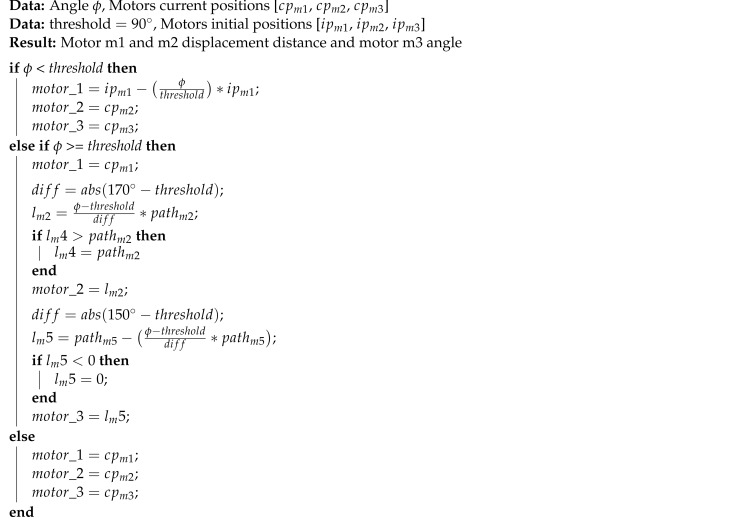

## Appendix D

Rehabilitation is controlled by the interface that sends the movement commands to the microcontroller. Different threads are thrown to monitor and create the commands. As shown at the Algorithm A3, the number of repetitions leads the rehabilitation and can be stopped at any time by pressing the stop button on the interface, making the robot move to the starting position.

**Algorithm A3:** State machine algorithm for shoulder movement
